# Nutrient Deficiency Tolerance in Citrus Is Dependent on Genotype or Ploidy Level

**DOI:** 10.3389/fpls.2019.00127

**Published:** 2019-02-11

**Authors:** Julie Oustric, Raphaël Morillon, François Luro, Stéphane Herbette, Paul Martin, Jean Giannettini, Liliane Berti, Jérémie Santini

**Affiliations:** ^1^CNRS, Laboratoire Biochimie and Biologie Moléculaire du Végétal, UMR 6134 SPE, Université de Corse, Corsica, France; ^2^Equipe “Amélioration des Plantes à Multiplication Végétative”, UMR AGAP, Département BIOS, CIRAD, Petit-Bourg, Guadeloupe; ^3^UMR AGAP Corse, Station INRA/CIRAD, San-Giuliano, France; ^4^UCA, INRA, PIAF, Clermont-Ferrand, France; ^5^AREFLEC, San-Giuliano, France

**Keywords:** antioxidant, citrus, nutrient deficiency, oxidative stress, photosynthesis, polyploid

## Abstract

Plants require essential minerals for their growth and development that are mainly acquired from soil by their roots. Nutrient deficiency is an environmental stress that can seriously affect fruit production and quality. In citrus crops, rootstock/scion combinations are frequently employed to enhance tolerance to various abiotic stresses. These tolerances can be improved in doubled diploid genotypes. The aim of this work was to compare the impact of nutrient deficiency on the physiological and biochemical response of diploid (2x) and doubled diploid (4x) citrus seedlings: Volkamer lemon, Trifoliate orange × Cleopatra mandarin hybrid, Carrizo citrange, Citrumelo 4475. Flhorag1 (*Poncirus trifoliata* + and willow leaf mandarin), an allotetraploid somatic hybrid, was also included in this study. Our results showed that depending on the genotype, macronutrient and micronutrient deficiency affected certain physiological traits and oxidative metabolism differently. Tetraploid genotypes, mainly Flhorag1 and Citrumelo 4475, appeared resistant compared to the other genotypes as indicated by the lesser decrease in photosynthetic parameters (*P*_net_, *F*_v_/*F*_m_, and *G*_s_) and the lower accumulation of oxidative markers (MDA and H_2_O_2_) in roots and leaves, especially after long-term nutrient deficiency. Their higher tolerance to nutrient deficiency could be explained by better activation of their antioxidant system. For the other genotypes, tetraploidization did not induce greater tolerance to nutrient deficiency.

## Introduction

Polyploidization is considered as a major force in plant evolution (Wendel, 2000; Chen, 2007; Soltis and Soltis, 2009). Polyploid organisms possess more than two sets of chromosomes. There are two different types of polyploidy: autopolyploids, which result from intraspecific genome duplication occurring during incomplete mitosis, or allopolyploids, which are formed from the combination of at least two complete chromosome sets via interspecific hybridization (Stebbins, 1947; Doyle and Egan, 2010). Polyploidy confers several advantages: genome buffering (mutation and recombination), allele-dosage effects, increased level of allelic diversity and heterozygosity, and sub- and/or neo-functionalization of duplicated genes that can induce phenotypic variation (Comai, 2005; Udall and Wendel, 2006; Beest et al., 2012). Polyploids are usually resistant or more tolerant compared to their progenitors to adverse environmental conditions such as extreme temperatures, high radiation levels and nutrient-poor soil (Ehrendorfer, 1980; Levin, 1983; Brochmann et al., 2004; Hao et al., 2013). Citrus are generally diploid, with 2n = 2x = 18 chromosomes (Krug, 1943); however tetraploids resulting from incomplete mitosis of somatic embryos are frequently found in seedlings of diploid apomictic genotypes (Cameron and Frost, 1968). Compared with the corresponding diploids, citrus 4x plants tend to have thicker and greener leaves, shorter thicker roots, larger fruit and depressed growth versus diploids (Ollitrault and Jacquemond, 1994; Guerra et al., 2014; Ruiz et al., 2016a,b,c). Anatomical and physiological changes between 4x and 2x counterparts (Syvertsen et al., 2000) could affect leaf mineral content.

Abiotic factors can cause oxidative stress due to an overproduction of ROS. An excess of ROS lead to protein denaturation, DNA mutation, and membrane lipid peroxidation (Mittler, 2002; Apel and Hirt, 2004; Mittler et al., 2004). It can be a tricky task to unravel the causes and the consequences in the oxidative metabolism responses. It implies to investigate both oxidative products levels like H_2_O_2_ as a primary marker of ROS and MDA as lipid peroxidation marker but also antioxidant responses with key antioxidant enzyme activities (mainly SOD, APX, CAT, and DHAR) and key antioxidant molecules such as ascorbate and proline. Antioxidant system has been found to be of paramount importance in the response and tolerance of trees to environmental stress (Polle et al., 1993; Mutlu et al., 2013; Santini et al., 2013; Xu et al., 2013).

In the citrus industry, one of the environmentally friendly techniques to prevent a drop in yield and fruit quality in abiotic stress conditions is grafting the scion onto a rootstock that confers better tolerance to those stresses. Rootstock selection is mainly based on the performance of the root system for coping with biotic (soil pathogens) and abiotic stress (salinity, drought, nutrient deficiency, and extreme temperature). Studies have demonstrated that using 4x seedlings or rootstocks enhances stress tolerance or allows to confer resistance when compared to their diploid counterparts (Saleh et al., 2008; Allario et al., 2013; Tan et al., 2015; Oustric et al., 2017).

When subjected to environmental stress, tolerant plants can offset the effects of oxidative damage while a resistant plant can prevent the rate of oxidative damage. These two strategies allow a plant protection from the harmful effects of stress (Mauricio et al., 1997).

Nutrient deficiency, particularly of a major nutrient (N, P, and K), is one of the most limiting factors along with drought, salinity, soil alkalinity, and extreme temperatures, on the performance of plants in their natural habitats and agricultural environments (Tewari et al., 2004). Plants require an optimal level of nutrients for growth and normal function. An excess has a negative effect on soil biology while scarcity has a negative impact on growth and development. In addition, nutrient deficiency can disturb the plant’s antioxidant system, as nutrients are needed for antioxidant biosynthesis (Kandlbinder et al., 2004). Plants possess a long distance mobile signal that allows them to respond to starvation and satiety signals throughout the plant through their vascular system. For example, the main symptoms of nutrient deficiency are stunted growth, chlorosis, necrosis and a disproportionately high allocation of biomass to the root system (Ericsson, 1995). Many genes play a central role in the acquisition and distribution of nutrients, including many protein-coding genes as well as microRNAs (miR395, miR399, miR398, miR397, and miR408) (Chiou, 2007; Sunkar et al., 2007).

In the citrus industry, nutrient deficiencies have an economic and ecologic importance. Thus, developing new citrus rootstocks that require less fertilizer is an urgent need, as well as improving our understanding of citrus responses to nutrient deficiency. The use of tetraploid rootstock could respond to this problem. However, to our knowledge, there is no information in the literature on the effect of genotype and ploidy level on resistance to nutrient deficiency. The aim of this study was to identify resistant or tolerant seedling genotypes among the most commonly used citrus rootstocks: VK, PMC, CC, CM. Moreover, in order to confirm the positive effect of polyploidization of the genotype on tolerance to nutrient deficiency, the 4x (doubled diploid) counterparts of these citrus seedlings were also studied. The allopolyploid Flhorag1 (FL)—a somatic hybrid of Trifoliate orange and Willow leaf mandarin—was also included in the experimental design (Ollitrault et al., 2000). The effects of nutrient deprivation and the plant’s tolerance were evaluated at the root and leaf levels by analyzing relevant anatomical, physiological, and biochemical parameters.

## Materials and Methods

### Plant Material and Growth Conditions

The experiment was performed on four citrus diploid (2x) seedling genotypes and their four doubled diploid (4x) counterparts (Table 1). The Flhorag1, an allotetraploid form, was also included. Plants were selected among seedlings made with seeds from trees maintained in the citrus germplasm collection (BCR NF 96-S-900 Citrus INRA/CIRAD) at San-Giuliano, Corsica (France). The ploidy status of 2x and 4x seedlings was checked by 10-color flow cytometry (Partec I, Germany) as described by Froelicher et al. (2007). Clonal propagation by nucellar embryogenesis was verified by genotyping using SSR markers as described by Vieira et al. (2016). The 12 seedlings of each genotype, giving a total of 108 plants were grown under identical conditions in vermiculite with ferti-irrigation and water (1 L/h) during 4 years in a tunnel greenhouse at the AREFLEC experimental station in San-Giuliano, Corsica (41°47′27″ N and 09°23′40″ E). The ferti-irrigation solutions were prepared and applied with a metering pump. The stock solution used for irrigation included 20-5-10 N (nitrogen) P (phosphorus) K (potassium) fertilizer + 2MgO (Magnesium oxide) + trace elements. This level of fertilization was based on the recommendations of the local department of agriculture. After 4 years and before starting the experiment, the vermiculite was washed with distilled water for 48 h in order to eliminate any nutritional reserves in the pot. This avoided the latency phase that could have occurred if the fertilizer remaining in the vermiculite had been completely consumed by the plants. The 4-years-old trees were then divided into two blocks: one with reference ferti-irrigation (control plants) and the other with irrigation water (without nutrient inputs). In each block, a total of three trees of each genotype was randomized, giving a total of 12 trees per block.

**Table 1:** Citrus varieties used for physiological and biochemical analysis.

			Ploidy	
Species	Varieties	Abbreviations	level	ICVN
*Citrus limonia* Osb.	Volkamer lemon	VK	2x	110024
			4x	101122
*Poncirus trifoliata* L. Raf. x *Citrus reshni* Hort. ex Tan.	Trifoliate orange x Cleopatra mandarin	PMC	2x	110155
			4x	101114
*Poncirus trifoliata* L. Raf. + *Citrus deliciosa* Ten.	Flhorag1	FL	4x	100951
*Citrus sinensis* L. Osb. *× Poncirus trifoliata* L. Raf.	Carrizo citrange	CC	2x	110181
			4x	101075
*Citrus paradisi* L. Macf. *× Poncirus trifoliata* L. Raf.	Citrumelo 4475	CM	2x	110410
			4x	101112

*ICVN, International Citrus Variety Numbering.*

This experiment was carried out from May 2016 to January 2017 on homogeneous plants comprising four branches with leaves developing under stress conditions and having reached full maturity. Nutrient deficiency was applied for 210 days and then trees were returned to the baseline condition with the control fertigation solution.

The samplings and physiological measurements were carried out at different times (days) selected based on preliminary experiments: 0 (control plant), 70 (D70), 140 (D140), and 210 (D210) days after the start of nutritional deprivation and after 30 days of recovery (30DR). For physiological measurements, nine fully expanded leaves were analyzed, i.e., three per tree (nine replicates), between 7 AM and 11 AM. For biochemical analyses, three samples were collected for each genotype, i.e., one per tree, and each sample is obtained by pooling eight fully-expanded leaves.

Root samplings for the biochemical measurements were performed at four different times: 0 (control plant), D70, D210, and 30DR. The trees were removed from their pots and cleared of residual vermiculite, and then rinsed with water. For the biochemical analysis, three samples of primary and secondary roots were collected at the same time for each genotype, i.e., one per tree (three replicates).

Mineral analyses were performed at three different times: 0 (control plant), D210 and 30DR. For the mineral measurements, three samples were collected for each genotype, i.e., one per tree, and each sample is obtained by pooling eight fully-expanded leaves (three replicates).

All leaves and roots were immediately frozen in liquid nitrogen and stored at -80°C, and each leaf sample was ground to a fine powder prior performing biochemical and mineral analysis.

Branches length, trunk diameter and leaf area were measured at four different times: 0 (control plant), D70, D210, and 30DR. Branches length was measured from the top of the trunk to the top of the highest leaf for four branches of each genotype, i.e., 12 per tree (12 replicates). Trunk diameter was measured using a caliper 5 cm from the soil surface for each genotype (three replicates). For leaf area, three fully expanded leaves for each genotype, i.e., three per tree, were selected and analyzed using ImageJ imaging software, version 1.47 (nine replicates).

### Evaluation of Leaf Damage

The genotypes were visually ranked according to the degree of nutrient deficiency damage after 210 days of nutrient deficiency. Nutrient deficiency damage (chlorosis, shriveled leaves) was evaluated on fully expanded leaves and scored using the following scale: level 0, no visible signs; level 1 – light green leaves with yellow leaves; level 2 – yellow leaves; level 3 – yellow shriveled leaves. The level of nutrient deficiency damage was evaluated in 15 independent leaves for each genotype.

### Foliar Mineral Analysis

All ground samples were dried at 65 ± 10°C in an oven overnight, transferred into a desiccator until cooling and sent into a CIRAD laboratory (Montpellier, France) for analysis of macro- and micro-nutrients.

Total phosphorus (P), potassium (K), calcium (Ca), magnesium (Mg), sodium (Na), bore (B), copper (Cu), iron (Fe), zinc (Zn), and manganese (Mn) in leaves were measured using an Agilent 720 simultaneous ICP-OES after double calcination including silica removal by adding hydrofluoric acid.

Nitrogen (N) content in leaves was determined by combustion using a Leco TruMac N determinator.

### Measurements of Gas Exchange

Leaf net photosynthetic rate (*P*_net_) and stomatal conductance (*G*_s_) were determined using a portable photosynthesis system (LI6400, Li-COR, Lincoln, NE, United States) with the LI6400-40 Leaf Chamber. For the *P*_net_ and *G*_s_ measurements, carbon dioxide concentration (CO_2_) was fixed at 380 μmol mol^-1^, airflow rate at 500 μmol s^-1^ and temperature at 25°C. Photosynthetic photon flux density was provided by a red-blue light source (6400-02B no. SI-710, Li-COR, Lincoln, NE, United States) in a gas exchange chamber and was set at 1400 μmol m^-2^ s^-1^.

### Measurements of Chlorophyll *a* Fluorescence

Chlorophyll *a* fluorescence was detected using a leaf fluorimeter (Handy PEA, Hansatech, Instruments, Ltd.) with a resolution of 10 s, in a non-destructive and reproducible manner. Leaves were adapted to the dark with a leaf clip for 30 min before the measurements were started. We measured the minimum value of chlorophyll *a* fluorescence (*F*_0_) that resulted in opening of all photosystem II (PSII) centers [oxidization of all the primary quinone acceptors (QA)] and the maximum fluorescence (*F*_m_) that results in closing of PSII centers (reduction of all QA) after the emission of a saturating 1-s flash of light (650 nm). Values of variable fluorescence (*F*_v_ = *F*_m_ -*F*_0_) and the maximum quantum efficiency of PSII primary photochemistry (*F*_v_/*F*_m_ [= (*F*_m_ -*F*_0_)/*F*_m_]) were calculated (Maxwell and Johnson, 2000).

### Evaluation of Leaf Damage

The genotypes were visually ranked according to the degree of nutrient deficiency damage after 210 days of nutrient deficiency. Nutrient deficiency damage (chlorosis, shriveled leaves) was evaluated on fully expanded leaves and scored using the following scale: level 0, no visible signs; level 1 – light green leaves with yellow leaves; level 2 – yellow leaves; level 3 – yellow shriveled leaves. The level of nutrient deficiency damage was evaluated in 15 independent leaves for each genotype.

### Determination of Oxidative Stress and Antioxidant Levels

Oxidative markers (malondialdehyde and hydrogen peroxide) assays in roots and leaves and ascorbate and antioxidant enzyme (superoxide dismutase, catalase, ascorbate peroxidase, and dehydroascorbate reductase) in leaves were performed as described by Santini et al. (2013). A V-630 spectrophotometer was used for all measurements (Jasco, Inc., Tokyo, Japan).

Proline content was measured in leaves using the ninhydrin reaction described by Bates et al. (1973), with slight modifications. Proline was extracted from 20 mg of leaf powder, suspended in 2 mL of sulfosalysilic acid 3% and then centrifuged at 11,500 *g* for 15 min at 4°C. The reaction mixture contained 600 μL of glacial acetic acid, 600 μL of ninhydrin acid (1.25 g of ninhydrin, 30 mL of glacial acetic acid and 20 mL of orthophosphoric acid 6 M) and 300 μL of supernatant. After 1 h of incubation at 95°C, 2 mL of toluene was added to extracted red products and samples were vortexed for 15 s. The absorbance of the organic phase was measured at 520 nm. Proline content was calculated using a standard curve of proline. A V-630 spectrophotometer was used for all measurements (Jasco, Inc., Tokyo, Japan).

### Statistical Analyses

All statistical measurements were performed with R statistical software^1^. The qualitative factors studied are sampling date (days) (D0, D70, D140, and D210 after nutrient deficiency, and after 30DR of recovery for leaves and D0, D70, and D210, and 30DR after recovery for roots), genotypes subjected to nutrient stress (VK, PMC, FL, CC, and CM for leaves and roots) and ploidy level nutrient stressed genotypes (VK2x/4x, PMC2x/4x, FL4x, CC2x/4x, CM2x/4x for leaves and roots). The influence of these three factors was analyzed using a two-way ANOVA followed by LSD test at *P* < 0.05. Heat map was made to determine the differences between genotypes, ploidy level and treatments for total content in macro- and micro-nutrients and growth parameters.

The data obtained at D0, D70, D140, and D210 of nutrient deficiency and after 30DR of recovery for the nine genotypes were analyzed by principal component analysis (PCA) of centered and reduced variables with a FactomineR package bundled with R statistical software. PCA was conducted to define a clear relationship between physiological (*P*_net_, *G*_s_, and *F*_v_/*F*_m_ ratio) and biochemical (oxidative status, antioxidant enzymes, and non-enzymatic molecules) parameters and genotypes after different periods of total nutrient deficiency and after 30 days of recovery. PCA contributed to better understanding of similarities between many measured variables and individuals.

## Results

In order to minimize any effect of environmental condition changes (photoperiod, temperature in the tunnel greenhouse, etc.) during the experiments, results on stressed seedlings were expressed as ratios relative to the values obtained on control seedlings. Thus, only the effect of the nutritional deprivation was taken into account. After 30 days of recovery (30DR), PMC2x and PMC4x lost all their leaves; consequently, the physiological and biochemical parameters were not measured.

### Leaf Damage and Growth Parameters

**FIGURE 1:**
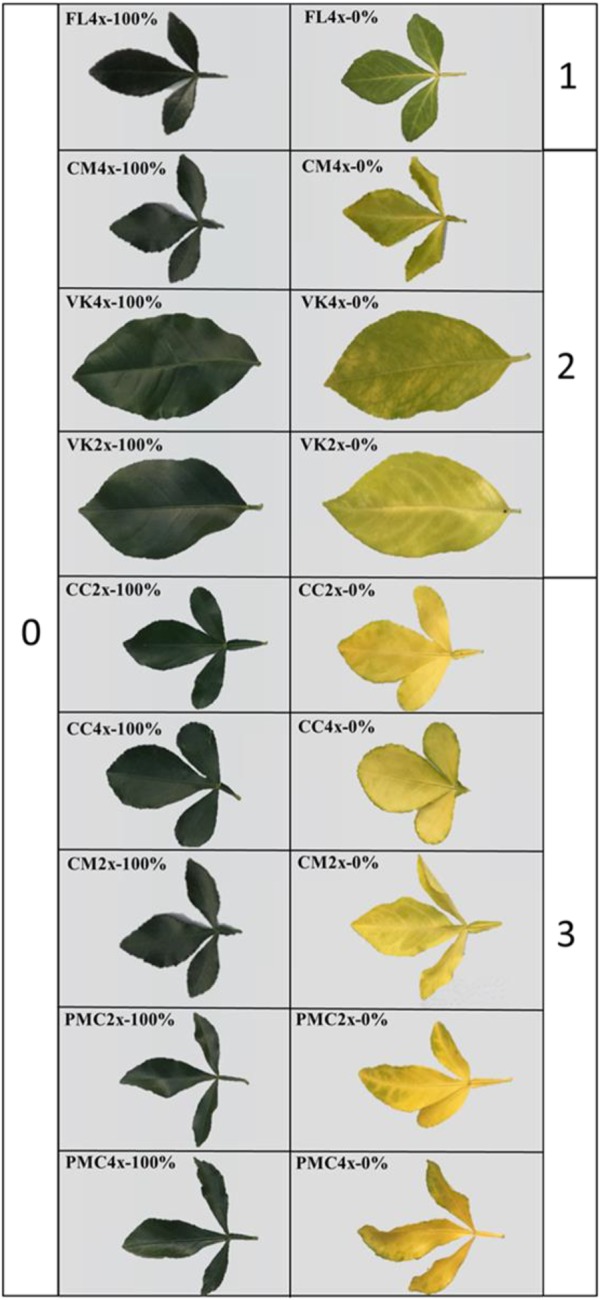
Leaf damages after 210 days of total nutrient deficiency on the nine genotypes (0%) compared to controls (100%). Genotypes are ranked based on the leaf symptoms from the unaffected (0) to the more affected (3).

Leaf damage signs were analyzed after 210 days of nutrient stress and were ranked using a four-level system based on the color of the leaves and veins (Figure 1). The level 0 corresponded to the control with green leaves. The least leaf damage (level 1) was found in FL4x with light green leaves. CM4x and VK2x and VK4x had very light green leaves with yellow veins and were classified as level 2. CC2x and CC4x, CM2x and PMC2x and PMC4x had yellow leaves and were classified as level 3. Overall, branches length, trunk diameter, and leaf area continued to grow after 210 days of nutrient deprivation and at after 30 days of recovery in all genotypes (Supplementary Table S1). Only FL4x and PMC4x did not show an increase in their leaves between D0 and D210 and D30DR (Supplementary Table S1).

### Change in Macro- and Micro-Nutrients

Overall after 210 days of nutrient stress all genotypes showed a significant decrease in N, Fe, Mn, Zn, and B in their leaves and a slight decrease or values close to the control in P, Mg, Ca, and Na. Only K remained substantially similar to the control for all genotype (Supplementary Table S1).

At 30DR, N, P, Ca, Na, Fe, Mn, Cu, Zn, and B contents were lower than the control values in all genotypes (Table 2 and Supplementary Table S2). Conversely, K and Mg contents fluctuated differently according to the genotypes (Table 2 and Supplementary Table S2). K content increased in VK2x and VK4x, FL4x and CC2x while Mg content increased in FL4x, CC2x and CC4x and CM2x and CM4x. In VK2x and VK4x and PMC2x and PMC4x, K and Mg contents decreased (Table 2 and Supplementary Table S2).

**Table 2:** Total content of nitrogen (N), phosphorus (P), potassium (K), magnesium (Mg), calcium (Ca) and sodium (Na), throughout the nutrient deficiency in leaves of 2x and 4x genotypes.

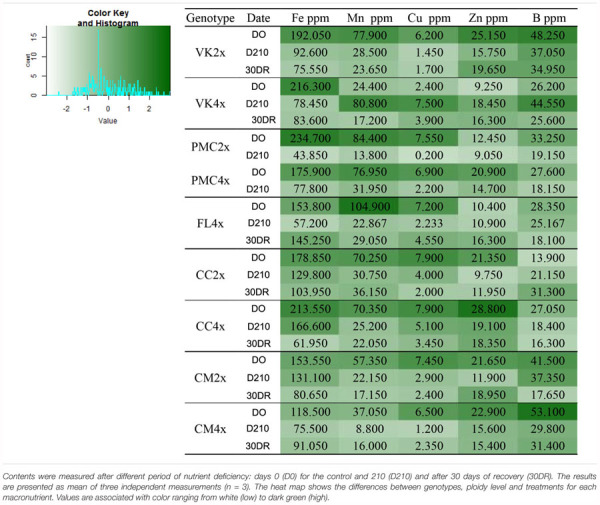

### Change in Photosynthetic Capacities

**FIGURE 2:**
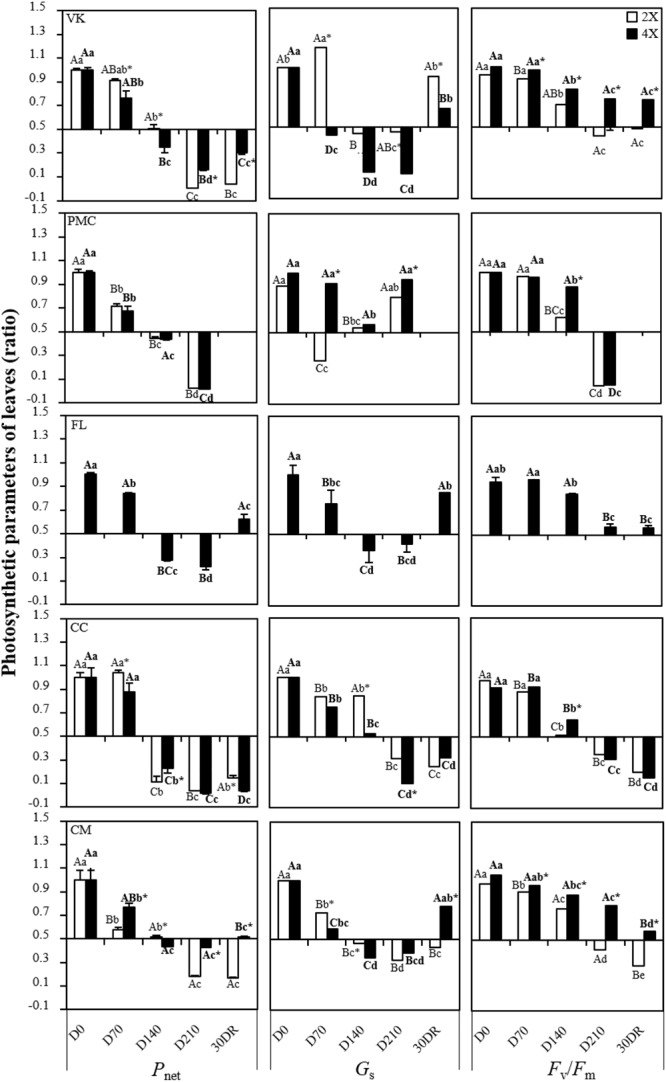
Evolution of net photosynthesis rate (*P*_net_), stomatal conductance (*G*_s_) and chlrorophyll fluorescence ratio (*F*_v_/*F*_m_), throughout the nutrient deficiency in leaves of 2x and 4x seedlings. The white and black bars correspond to the values obtained from 2x and 4x seedlings, respectively.*(Concentrations were measured after different period of nutrient deficiency: days 0 (D0) for the control, 70 (D70), 140 (D140) and 210 (D210) and after 30 days of recovery (30DR). The results obtained are expressed as ratios with respect to the values obtained on control leaves which have not been subjected to stress. The results are presented as mean (± standard error) of nine independent measurements (*n* = 9). Data were analyzed using ANOVA and Fisher LSD tests (*P* < 0.05). Distinct capital letter indicate significant differences between all diploid seedlings at a point of the time course. Different lower case letters indicate significant value change along the time course for in 2x seedlings. For 4x seedlings, the same procedure has been followed and the results are indicated in bold roman. An asterisk indicates significant differences between 2x and 4x seedlings of the same variety at a point of the time course.)*

*P*_net_ begun to decrease at D70 in VK2x and VK4x, PMC2x and PMC4x, FL4x and CM2x and CM4x and from D140 in CC2x and CC4x (Figure 2).

This was generally associated with a decrease in *G*_s_ (Figure 2). At D210, FL4x, and CM4x had the lowest decrease both in *P*_net_ and *G*_s_. Interestingly, *G*_s_ increased at D210 in PMC2x and PMC4x while *P*_net_ continued to decline.

*F*_v_/*F*_m_ decreased early on at D70 in PMC2x and CM2x and CM4x, and at D140 in VK2x and VK4x, PMC4x, FL4x and CC2x and CC4x (Figure 2). However, at D210 and 30DR, *F*_v_/*F*_m_ was greater in FL4x, CM4x, and VK4x than in the other genotypes.

### Change in Oxidative Markers (MDA and H_2_O_2_) in Leaves and Roots

**FIGURE 3:**
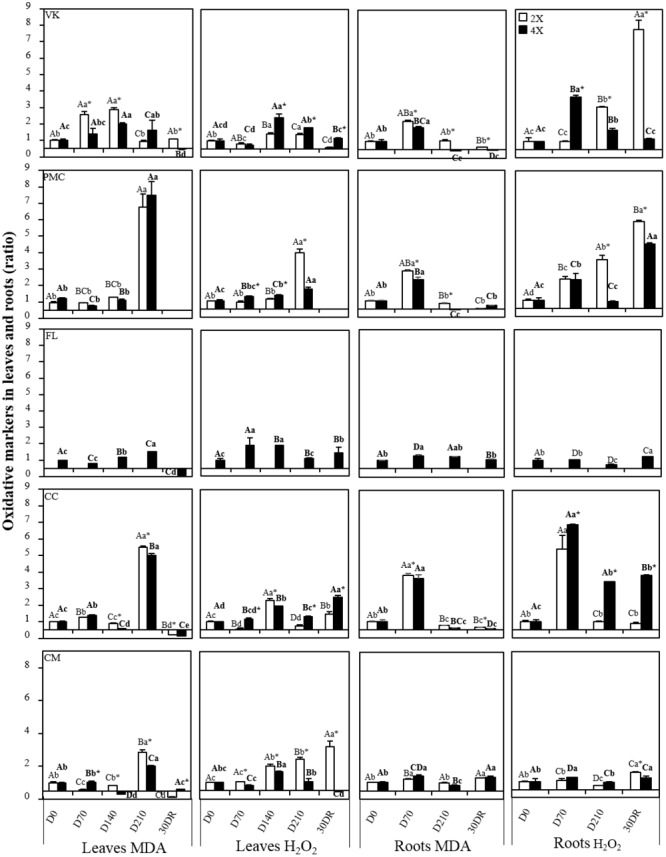
Evolution of malondialdehyde (MDA) and hydrogen peroxide (H_2_O_2_) concentration, throughout the nutrient deficiency in leaves and roots of 2x and 4x genotypes. The white and black bars correspond to the values obtained from 2x and 4x genotypes, respectively. Concentrations were measured after different period of nutrient deficiency in: days 0 (D0) for the control, 70 (D70), 140 (D140) and 210 (D210) and after 30 days of recovery (30DR) in leaves and days 0 (D0) for the control, 70 (D70) and 210 (D210) and after 30 days of recovery (30DR) in roots. The results obtained are expressed as ratios with respect to the values obtained, respectively, on control leaves and control roots which have not been subjected to stress. The results are presented as mean (± standard error) of three independent measurements (*n* = 3). Data were analyzed using ANOVA and Fisher LSD tests (*P* < 0.05). Distinct capital letter indicate significant differences between all 2x genotypes at a point of the time course. Different lower case letters indicate significant value change along the time course for one 2x genotype. For 4x genotypes, the same procedure has been followed and the results are indicated in bold roman. An asterisk indicates significant differences between 2x and 4x genotypes of the same variety at a point of the time course.

Overall, MDA increased lately in leaves for all genotypes, excepted in VK2x (Figure 3). At D210, PMC2x and PMC4x, CC2x and CC4x and CM2x had a peak of MDA accumulation, while there was a slight increase in FL4x and PMC4x and a decrease in VK2x. After 30 days of recovery, MDA returned to the same or a lower level than in all control genotypes. H_2_O_2_ increased early on FL4x, but decreased to the same values as the control at D210 (Figure 3). H_2_O_2_ increased from D140 in VK2x and VK4x, PMC4x, CC2x and CC4x and CM2x and CM4x and from D210 in PMC2x. Like for FL4x, H_2_O_2_ decreased at D210 in CC2x. At 30DR, unlike MDA, H_2_O_2_ values were higher than for all control genotypes, excepted in CM4x.

Measurements of H_2_O_2_ and MDA contents in roots throughout the experiment revealed no large changes induced by nutrient deficiency in FL4x and CM2x and CM4x, contrary to the other genotypes (Figure 3). MDA increased early on VK2x and VK4x, PMC2x and PMC4x and CC2x and CC4x, but decreased at D210 and after 30 days of recovery (30DR). H_2_O_2_ content also increased early on VK4x, PMC2x and PMC4x and CC2x and CC4x but decreased in VK4x, PMC4x, and CC2x after D210.

### Change in Enzymatic Antioxidants

Overall, SOD and DHAR increased or were similar to control in all genotypes (Supplementary Figure S1). SOD decreased in CM2x only throughout the deficiency period. CAT decreased in VK2x and VK4x, PMC2x and PMC4x and CC2x and CC4x, whereas it increased from D140 in FL4x (Supplementary Figure S1). APX remained stable during the deficiency period in VK2x and VK4x whereas it was higher from D140 compared to control in PMC2x and PMC4x, FL4x, CC2x and CC4x and CM2x and CM4x. However, PMC2x decreased at D210 (Supplementary Figure S1).

### Change in Antioxidant Molecules

The tAsa, Asa and DHA contents increased in all genotypes during the nutrient deficiency period and after the recovery period (Supplementary Figure S2). The Asa/DHA ratio decreased from D70 in FL4x and CC2x and from D140 in VK2x and VK4x, PMC2x and PMC4x, CC4x and CM2x and CM4x (Supplementary Figure S2). However, FL4x and CM4x had an increased Asa/DHA ratio at D210. At 30DR, the Asa/DHA ratio decreased in all genotypes.

The proline content decreased as early as D70 in all genotypes, but to a lesser extent in FL4x. At 30DR, proline increased but did not reach values similar to the controls (Supplementary Figure S3).

### Responses of Citrus Genotypes Evaluated Through Physiological and Chemical Parameters During the Time Course of the Stress

**FIGURE 4:**
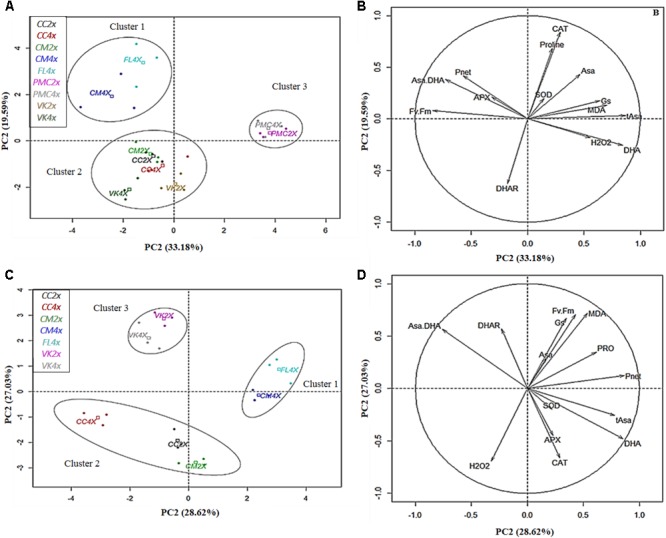
Biplot obtained from PCA performed after **(A,B)** 210 days of total nutrient deficiency and 30 days of recovery **(C,D)** in leaves of the nine genotypes. **(A,C)** dispersion of genotypes and **(B,D)** contribution of the variables to the dispersion. The variables analyzed are net photosynthesis rate (*P*_net_), stomatal conductance (*G*_s_), chlorophyll fluorescence (*F*_v_/*F*_m_), malondialdehyde (MDA), hydrogen peroxide (H_2_O_2_), antioxidant enzymes (SOD, CAT, APX, and DHAR), reduced ascorbate (Asa), oxidized ascorbate (DHA), total ascorbate (tAsa), ascorbate redox status (Asa/DHA), and proline.

Based on the PCA, the first two principal axes explained 49.84, 49.55, and 52.77% for 70 (D70), 140 (D140) (Supplementary Figure S4) and 210 days (D210) after nutrient deficiency, respectively, and 55.65% for 30 days after recovery (30DR) (Figure 4). Genotypes were already split in three distinct groups based on their response to nutrient deficiency. However, genotypes showed clear differences in signs of nutrient deficiency only at D210 (Figure 1).

Interestingly, at D210, PC1 was positively correlated with oxidative markers (MDA, H_2_O_2_, and DHA) and negatively with photosynthetic parameters (*P*_net_ and *F*_v_/*F*_m_ ratio) and Asa/DHA ratio. PC2 was positively correlated with CAT and proline, and negatively correlated with DHAR. PC1 separated cluster 1 (FL4x and CM4x) from cluster 3 (PMC2x and PMC4x) and PC2 separated cluster 2 (CC2x and CC4x, VK2x and VK4x and CM2x) from cluster 1. Thus, FL4x and CM4x were differentiated by low oxidative marker accumulation (MDA, H_2_O_2_, and DHA) and a higher photosynthetic capacity than in PMC2x and PMC4x. While CC2x and CC4x, VK2x and VK4x and CM2x had intermediate values for the variables compared to FL4x and CM4x, and PMC2x and PMC4x. These findings in FL4x and CM4x were associated with a higher Asa/DHA ratio and higher CAT in FL4x and APX in CM4x.

After 30DR, PC1 was positively correlated with photosynthetic parameters (*P*_net_, *G*_s_, and *F*_v_/*F*_m_ ratio), proline, tAsa and DHA, and negatively correlated with Asa/DHA. PC2 was positively correlated with *G*_s_, *F*_v_/*F*_m_ ratio, Asa/DHA, DHAR and MDA, and negatively correlated with DHA, H_2_O_2_, CAT, and APX. PC1 separated cluster 1 (FL4x and CM4x) from cluster 2 (CC2x and CC4x and CM2x) and cluster 3 (VK2x and VK4x) and PC2 separated cluster 2 from the cluster 3. Therefore, FL4x and CM4x were characterized by higher *P*_net_, *G*_s_, and *F*_v_/*F*_m_ ratio and proline, and lower H_2_O_2_ than CC2x and CC4x and CM2x, while VK2x/VK4x had intermediate values for these variables. VK2x and VK4x were distinguished by a high Asa/DHA ratio and high DHAR activity but lower SOD, CAT, and APX activities than the other genotypes.

## Discussion

### Comparison of the Sensitivity to Nutrient Deficiency of Citrus Seedling Genotypes

All the genotypes showed leaf damage after a long period of nutrient deficiency (Figure 1). This leaf damage suggests a decrease in the quantity of photosynthetic pigments and thus photosynthetic capacity. This is typical of leaf senescence under nutrient deprivation (Deng et al., 2001; Boussadia et al., 2010). Leaf damage was associated with an alteration of foliar mineral content. This indicates that our experimental conditions were suitable for screening genotypes for nutrient deficiency. Leaf damage is a visual sign used to rank genotypes depending on the impact of the nutrient deficiency (Srivastava, 2013). In our study, we associated them with the levels of oxidative markers and macronutrient contents at the same time in order to propose a precise classification (Figure 3 and Table 2) which will be checked later with the photosynthetic capacity and the antioxidant responses (Figure 2, 4 and Supplementary Figures S1, S4). To our knowledge, there is no information about the impact of nutrient deficiency in the citrus seedling genotypes studied in this experiment.

Level 3 of leaf damage, the significant depletion of leaf macronutrient and the large accumulation of MDA in CC2x and CC4x and PMC4x, and MDA and H_2_O_2_ in PMC2x and CM2x, means these genotypes are sensitive to nutrient deficiency. CM4x and VK2x and VK4x were ranked as potentially tolerant because their leaves had level 2 damage, significant macronutrient depletion and low MDA and H_2_O_2_ accumulation. Finally FL4x was classified as resistant because its leaves had level 1 damage and lo MDA and H_2_O_2_ accumulation. It was the only one to avoid the depletion of macronutrients.

In our study, the effect of autotetraploidization did not systematically improve the tolerance to nutrient deficiency. CM4x was the only 4x seedling with a significantly different response to nutrient deficiency compared to its 2x counterpart. This in agreement with previous results showing that in salt stress conditions, CM4x was more tolerant than CM2x (Podda et al., 2013). On the other hand, results in FL4x—which was the only resistant genotype—suggest that allotetraploidization would be a mean for increasing tolerance to nutrient deficiency. This needs to be supported by additional tests on other allotetraploids (Mouhaya et al., 2010). Chromosome doubling can increase tolerance to stress, have no effect or be harmful to the plant (Comai, 2005). For example, Liu et al. (2011) showed that drought, cold and salt tolerance in 4x *Dendranthema nankingense* were improved via chromosome doubling, but heat tolerance was not. In allopolyploid species, enhanced tolerance was more frequently observed than their parental species (Aïnouche et al., 2009).

### What Traits Are Related to Resistance to Nutrient Deficiency in Citrus Genotypes?

The “resistant” genotype, FL4x, and also the “moderately tolerant” genotype, CM4x, belonged to the same cluster (cluster 1) (Figure 4A), despite different levels of leaf damage and macronutrients content at D210. They appeared to have less disturbance of their photosynthetic process after D210 than the other genotypes as indicated by their higher *F*_v_/*F*_m_ ratio and *P*_net_ (Figure 4A,B). These results suggest a less marked photo-inhibition than in other genotypes probably due to better protection of their reaction centers of photosystem II (Huang et al., 2004). FL4x would be resistant while CM4x would be more tolerant than the genotypes of the group classified as potentially tolerant (VK2x and VK4x). Indeed, many studies have found a direct influence of nutritional stress on photosynthetic functions such as PSII photochemistry either in the electron transport chain or in a modified PSII structure (Baker and Rosenqvist, 2004; Molassiotis et al., 2006; Kalaji et al., 2014). Compared to the “moderately tolerant” VK2x and VK4x and the “sensitive” CC2x and CC4x and CM2x, which were in the same cluster at D210 (cluster 2) (Figure 4A), the smallest decrease in *P*_net_ was related to a lesser decrease in *G*_s_ (Figure 2), lower stomatal limitation and thus decreased CO_2_ flux that governs the Rubisco carboxylation sites in the chloroplast stroma (Flexas et al., 2013). The lesser decrease in *G*_s_ could also be due to a difference in the concentration of metabolites (sugars and organic acids) and a combination of stimuli that influences opening of the stomata (Boussadia et al., 2010; Lawson and Blatt, 2014; Gago et al., 2016). However, the “sensitive” PMC2x and PMC4x (cluster 3) (Figure 4A) were the only genotypes to show similar *G*_s_ values compared to control at D210. Opening of the stomata does not seem to result in the maintenance of photosynthesis in these genotypes during nutrient deficiency.

After 30 days of recovery, the high photosynthetic parameter values (*P*_net_, *G*_s_, and *F*_v_/*F*_m_) seem to indicate better recovery and therefore reversibility of the damage caused by nutrient deficiency in FL4x and CM4x compared to the other genotypes (Figure 4C,D). VK2x and VK4x had slightly lower recovery than FL4x and CM4x, but greater than CC2x and CC4x and CM2x (Figure 4C,D). At 30DR, no leaves resisted to the nutritional stress in PMC2x and PMC4x, which suggests a lack of recovery on injured leaves.

On the whole, the strongest photosynthetic capacities observed in FL4x and CM4x, and to a lesser extent in VK2x and VK4x than in CM2x, CC2x and CC4x and PMC2x and PMC4x could be explained by better accumulation and/or remobilization via the phloem transport of mineral elements related to photosynthesis activity (Marschner, 1995; Reich et al., 1999; Maillard et al., 2015) during nutrient deficiency. It could also be explained by a smaller decrease in transcript level related to essential components of photosynthesis (Wang et al., 2015).

### Antioxidant System Contributes to Improve Tolerance to Nutrient Deficiency in Both Roots and Leaves

During nutrient deficiency, enzymatic and non-enzymatic responses were variable and more or less effective depending on the genotype. The “resistant” FL4x and “tolerant” CM4x appeared to have a better performing non-enzymatic and enzymatic antioxidant system than the other genotypes. This helps to prevent the accumulation of toxic compounds after nutrient deficiency at D210. These results were consistent with less decrease in photosynthetic capacity (Figure 4A,B). Unlike the other genotypes, FL4x and CM4x had lower levels of oxidative markers (H_2_O_2_, MDA, and DHA) and higher Asa/DHA ratio (Figure 4A,B) and proline content (especially for FL4x; Supplementary Figure S3) at D210. At the root level, the least fluctuation of MDA and H_2_O_2_ in FL4x and CM4x was consistent with the tolerance found in their leaves during nutrient deficiency and after 30 days of recovery (Figure 3). All results obtained confirmed the classification as resistant and tolerant of these two genotypes. An increase in MDA and H_2_O_2_ at D70 was observed in other genotypes suggesting an early root response to oxidative stress in comparison to their leaves (Shin and Schachtman, 2004; Shin et al., 2005).

Regarding antioxidant enzyme systems, all genotypes had a higher or similar value in SOD and DHAR activities when compared to controls (Supplementary Figure S1). However, different variation of APX and CAT activities (Supplementary Figure S1) were observed. At D210, CM4x had the greatest APX activities whereas FL4x had the greatest CAT activities (Figure 4A,B), suggesting a difference in their H_2_O_2_ scavenging mechanism during nutrient deficiency (Gill and Tuteja, 2010). When subjected to salt stress, CM4x was more tolerant than its respective CM2x, which was correlated with higher constitutive levels of antioxidant enzymes and heat shock proteins (Podda et al., 2013). High APX activity was also found at D210 in “moderately tolerant” VK2x and VK4x and in the “sensitive” CM2x and CC2x/CC4x (Figure 4A,B). No association was found with all the antioxidant enzymes in the other “sensitive” PMC2x and PMC4x, confirming the importance of an efficient antioxidant enzyme system for tolerance to nutrient deficiency.

In FL4x and CM4x, the lower H_2_O_2_ and MDA levels can be explained by their higher or similar CAT and APX activities at D140 and D210 responsible for removing H_2_O_2_ formed by SOD, respectively (Azevedo et al., 1998). When excess H_2_O_2_ is not eliminated by CAT and APX, high levels of hydroxyl radical production may be responsible for lipid membrane damage. This damage is highlighted by the accumulation of MDA observed at D210 (Figure 3) in CC2x and CC4x, CM2x and PMC2x and PMC4x. The high DHAR activity in all genotypes did not prevent the accumulation of DHA, especially at D210 (Supplementary Figure S2). Only FL4x and CM4x showed maintenance of Asa/DHA near the control levels at D210 (Supplementary Figure S2). This could be attributable to *de novo* synthesis of ascorbate. The large increase in DHA (Supplementary Figure S2) may indicate a key role of ascorbate during nutritional deprivation. Functional collaboration between the different enzymes and antioxidant molecules seems to be crucial for an effective antioxidant system (Blokhina et al., 2003).

During recovery, specific behaviors of antioxidant machinery were observed between “resistant,” “tolerant,” “moderately tolerant,” and “sensitive” genotypes. CC2x and CC4x and CM2x had very high H_2_O_2_ accumulations which was consistent with the large disturbance in photosynthetic capacity, comparatively to the other genotypes (Figure 4C,D). In FL4x and CM4x, the better recovery was probably due to the higher activity of their non-enzymatic (proline and Asa), and to a lesser extent enzymatic (SOD, CAT, and APX), systems (Figure 4C,D).

## Conclusion

Our study used experimental design conditions that revealed differences between Citrus genotypes in terms of their resistance to nutrient deficiency. Future studies can be performed to improve citrus production in reduced input conditions. Using this experimental set up, the next steps would be to assay the mineral elements in plant organs for these different genotypes, to test the effects on growth and fruit production and quality.

This study demonstrated that total nutrient deficiency impairs the photosynthetic capacity as well as the oxidative metabolism of all citrus genotypes in different proportions. Nutrient deficiency highly affects the photosynthesis efficiency in sensitive genotypes by strong stomatal limitation and alteration of photosystem II. During nutrient deficiency and after recovery, the resistant FL4x and tolerant CM4x were the only genotypes characterized by lower accumulation of oxidative markers (MDA and H_2_O_2_) in leaves and roots due to a more effective antioxidant system. However, chromosome doubling does not improve tolerance in the other genotypes. The fact that these two 4x genotypes limit the damage caused to plant growth during nutrient deficiency suggests they can be used in citrus orchards for their horticultural performance as rootstock. This would reduce the amount of fertilizer needed and have a positive impact on soil biology while preserving growth. It would be interesting to confirm these results by testing the rootstocks/scion combination in order to evaluate whether use of these 4x rootstocks can improve the tolerance to nutrient deficiency of the scion. The impact of these combinations on growth, fruit production and fruit quality of the scion during nutrient deficiency should also be studied.

## Author Contributions

JO collected the test data, performed the statistical analyses, interpreted the results, and drafted the manuscript. JS interpreted the results and drafted the manuscript. LB, JG, SH, FL, and RM helped to draft the manuscript. PM helped to collect the test data.

## Conflict of Interest Statement

The authors declare that the research was conducted in the absence of any commercial or financial relationships that could be construed as a potential conflict of interest.

## Footnotes

1 http://www.R-project.org

## Abbreviations

2x: diploid
4x: tetraploid
APX: ascorbate peroxidase
Asa: reduced ascorbate
Asa/DHA: redox status of ascorbate
CC2x: diploid Carrizo citrange
CC4x: tetraploid Carrizo citrange
CAT: catalase
CM2x: diploid Citrumelo 4475
CM4x: tetraploid Citrumelo 4475
DHA: oxidized ascorbate
*F*_0_: minimal chlorophyll *a* fluorescence
*F*_m_: maximal fluorescence
*F*_v_: variable fluorescence
*F*_v_/*F*_m_: maximum quantum efficiency of PSII
*G*_s_: stomatal conductance
MDA: malondialdehyde
PMC2x: diploid hybrid Trifoliate orange x Cleopatra mandarin
PMC4x: tetraploid hybrid Trifoliate orange x Cleopatra mandarin
*P*_net_: leaf net photosynthetic rate
ROS: reactive oxygen species
SOD: superoxide dismutase
tAsa: total ascorbate
VK2x: diploid Volkamer lemon
VK4x: tetraploid Volkamer lemon
