# Supplementary material for: Nutrient Deficiency Tolerance in Citrus Is Dependent on Genotype or Ploidy Level
Source: Front Plant Sci. 2019 Feb 11;10:127. doi: 10.3389/fpls.2019.00127 (PMC6396732; doi:10.3389/fpls.2019.00127)
Supplement: Supplementary file 1 [file Table_1.pdf]

Table S1. Evolution of branches length, trunk diameter and leaf area, throughout the nutrient deficiency in leaves of 2x and 4x seedlings.

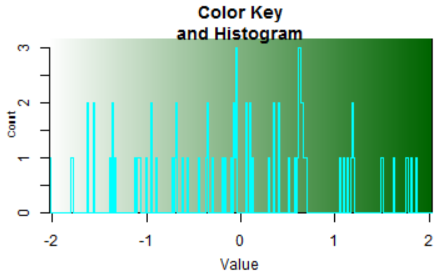

Genotype	Date	Branches length (cm)	Trunk diameter (cm)	Leaf area (cm)
VK2x	D0	27.00	17.53	6.33
	D210	58.96	21.02	10.07
	30DR	59.29	22.59	10.35
VK4x	D0	43.19	15.23	5.72
	D210	90.61	18.90	10.01
	30DR	91.20	18.60	10.79
PMC2x	D0	71.06	15.38	4.79
	D210	103.18	20.67	2.52
PMC4x	D0	52.98	13.60	4.02
	D210	92.24	14.75	5.60
FL4x	D0	43.19	12.95	3.23
	D210	90.61	14.07	2.52
	30DR	91.20	14.91	2.67
CC2x	D0	71.80	16.59	5.07
	D210	92.06	18.77	5.60
	30DR	104.70	18.77	5.56
CC4x	D0	74.74	17.94	6.56
	D210	118.54	19.51	6.72
	30DR	120.20	20.77	6.67
CM2x	D0	49.58	15.85	6.66
	D210	74.10	21.02	7.95
	30DR	74.39	21.00	7.97
CM4x	D0	49.44	16.16	7.66
	D210	74.06	18.90	7.83
	30DR	74.64	19.61	7.91

Growth parameters were measured after different period of nutrient deficiency: days 0 (D0) for the control and 210 (D210) and after 30 days of recovery (30DR). The results are presented as mean of 12 independent measurements ($n = 12$) for branches length and leaf area and 3 independent measurement ($n = 3$) for trunk diameter. The heat map shows the differences between genotypes, ploidy level and treatments for each growth parameters. Values are associated with color ranging from white (low) to dark green (high).
