# Supplementary material for: Nutrient Deficiency Tolerance in Citrus Is Dependent on Genotype or Ploidy Level
Source: Front Plant Sci. 2019 Feb 11;10:127. doi: 10.3389/fpls.2019.00127 (PMC6396732; doi:10.3389/fpls.2019.00127)
Supplement: Supplementary file 2 [file Table_2.pdf]

Table S2. Total content of iron (Fe), manganese (Mn), copper (Cu), zinc (Zn) and boron (B), throughout the nutrient deficiency in leaves of 2x and 4x genotypes.

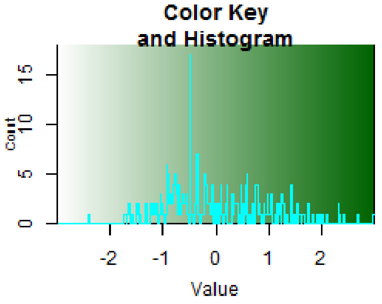

Genotype	Date	Fe ppm	Mn ppm	Cu ppm	Zn ppm	B ppm
VK2x	D0	192.050	77.900	6.200	25.150	48.250
	D210	92.600	28.500	1.450	15.750	37.050
	30DR	75.550	23.650	1.700	19.650	34.950
VK4x	D0	216.300	24.400	2.400	9.250	26.200
	D210	78.450	80.800	7.500	18.450	44.550
	30DR	83.600	17.200	3.900	16.300	25.600
PMC2x	D0	234.700	84.400	7.550	12.450	33.250
	D210	43.850	13.800	0.200	9.050	19.150
PMC4x	D0	175.900	76.950	6.900	20.900	27.600
	D210	77.800	31.950	2.200	14.700	18.150
FL4x	D0	153.800	104.900	7.200	10.400	28.350
	D210	57.200	22.867	2.233	10.900	25.167
	30DR	145.250	29.050	4.550	16.300	18.100
CC2x	D0	178.850	70.250	7.900	21.350	13.900
	D210	129.800	30.750	4.000	9.750	21.150
	30DR	103.950	36.150	2.000	11.950	31.300
CC4x	D0	213.550	70.350	7.900	28.800	27.050
	D210	166.600	25.200	5.100	19.100	18.400
	30DR	61.950	22.050	3.450	18.350	16.300
CM2x	D0	153.550	57.350	7.450	21.650	41.500
	D210	131.100	22.150	2.900	11.900	37.350
	30DR	80.650	17.150	2.400	18.950	17.650
CM4x	D0	118.500	37.050	6.500	22.900	53.100
	D210	75.500	8.800	1.200	15.600	29.800
	30DR	91.050	16.000	2.350	15.400	31.400

Contents were measured after different period of nutrient deficiency: days 0 (D0) for the control and 210 (D210) and after 30 days of recovery (30DR). The results are presented as mean of 3 independent measurements (*n* = 3). The heat map shows the differences between genotypes, ploidy level and treatments for each micronutrient. Values are associated with color ranging from white (low) to dark green (high).
