# Supplementary material for: Nutrient Deficiency Tolerance in Citrus Is Dependent on Genotype or Ploidy Level
Source: Front Plant Sci. 2019 Feb 11;10:127. doi: 10.3389/fpls.2019.00127 (PMC6396732; doi:10.3389/fpls.2019.00127)

Figure S1: Evolution of antioxidant enzyme specific activities: SOD, CAT, APX and DHAR, throughout the nutrient deficiency in leaves of 2x and 4x genotypes. The white and black bars correspond to the values obtained from 2x and 4x genotypes, respectively. Concentrations were measured after different period of nutrient deficiency: days 0 (D0) for the control, 70 (D70) and 210 (D210) and after 30 days of recovery (30DR). The results obtained are expressed as ratios with respect to the values obtained on control leaves which have not been subjected to stress. The results are presented as mean (± standard error) of 3 independent measurements (*n* = 3). Data were analysed using ANOVA and Fisher LSD tests ($P < 0.05$). Distinct capital letter indicate significant differences between all 2x genotypes at a point of the time course. Different lower case letters indicate significant along the time course for one 2x genotype. For 4x genotypes, the same procedure has been followed and the results are indicated in bold roman. An asterisk indicates significant differences between 2x and 4x genotypes of the same variety

at a point of the time course.

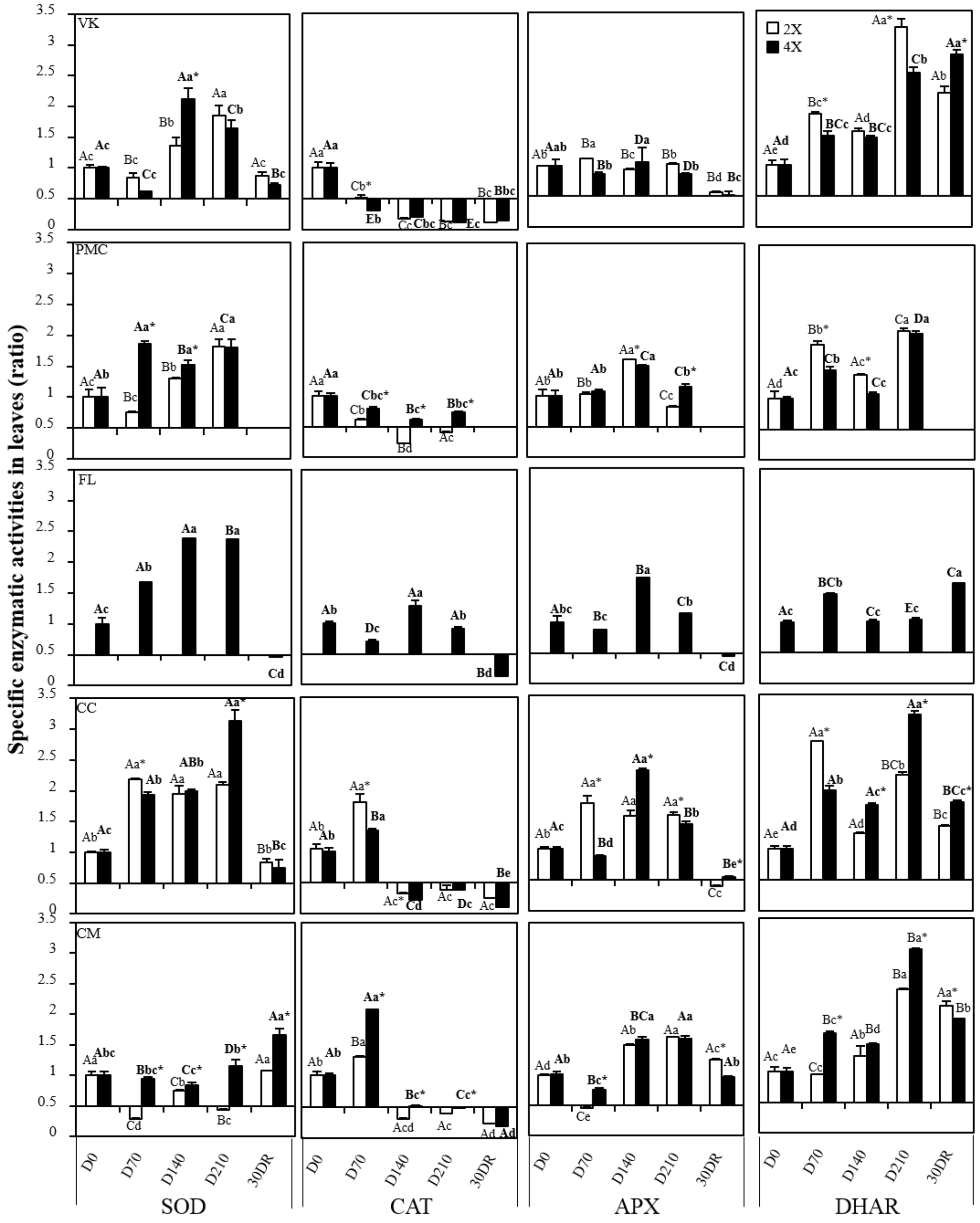

Supplement: Supplementary file 3 [file Image_1.pdf]
