# Supplementary material for: Nutrient Deficiency Tolerance in Citrus Is Dependent on Genotype or Ploidy Level
Source: Front Plant Sci. 2019 Feb 11;10:127. doi: 10.3389/fpls.2019.00127 (PMC6396732; doi:10.3389/fpls.2019.00127)
Supplement: Supplementary file 6 [file Image_4.pdf]

Figure S4: Biplot obtained from principal component analysis (PCA) performed after (A and B) 70 and (C and D) 140 days of total nutrient deficiency in leaves of the nine genotypes, (A and C) dispersion of genotypes and (B and D) contribution of the variables to the dispersion. The variables analyzed are net photosynthesis rate (P_{net}), stomatal conductance (G_s), chlorophyll fluorescence (F_v/F_m), malondialdehyde (MDA), hydrogen peroxide (H_2O_2), antioxidant enzymes (SOD, CAT, APX and DHAR), reduced ascorbate (Asa), oxidized ascorbate (DHA), total ascorbate (tAsa), ascorbate redox status (Asa/DHA) and proline.

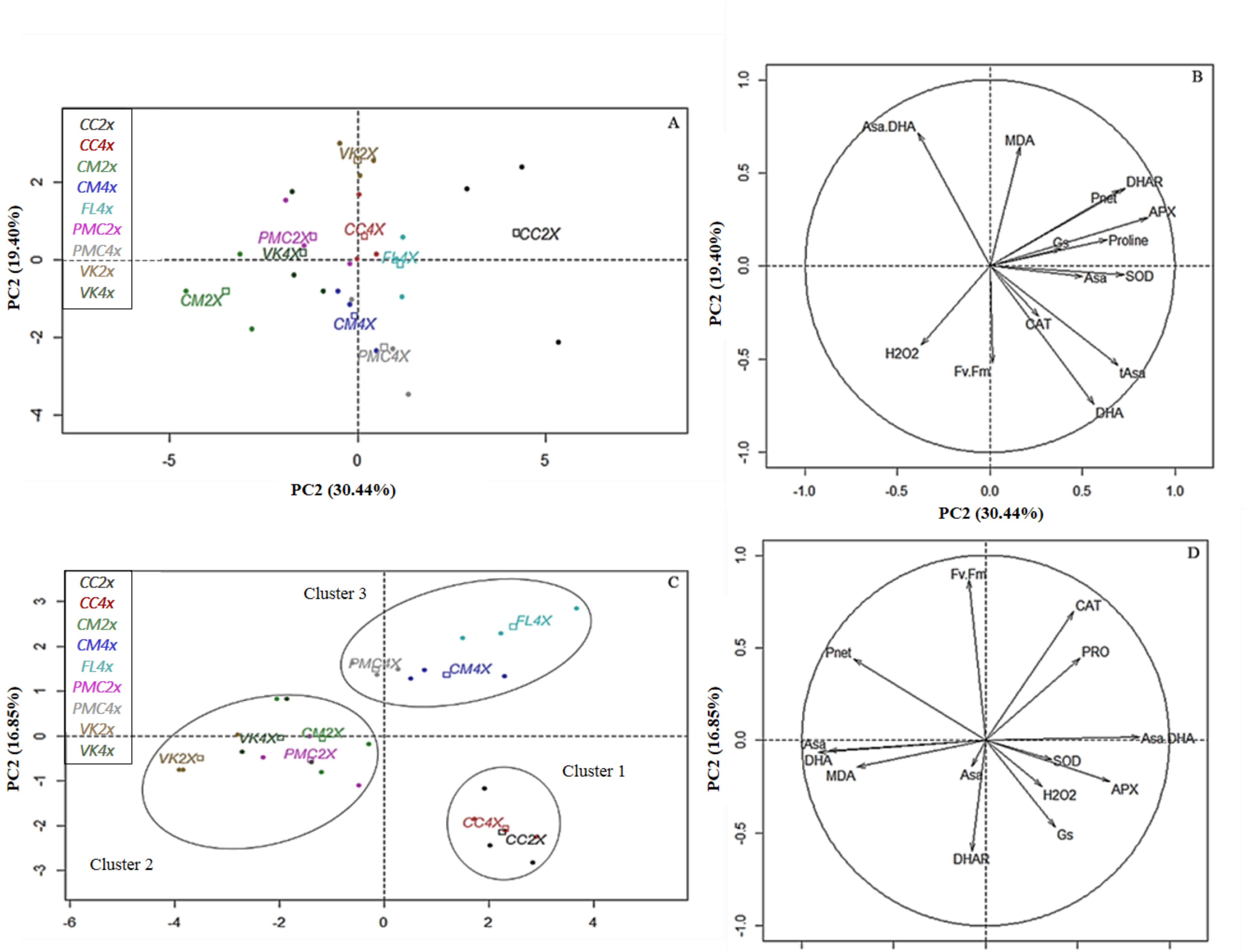
A
B
C
D
CC2x
CC4x
CM2x
CM4x
FL4x
PMC2x
PMC4x
VK2x
VK4x
VK2X
CC4X
CC2X
PMC2X
VK4X
FL4X
CM2X
CM4X
PMC4X
PC2 (19.40%)
PC2 (30.44%)
PC2 (16.85%)
Asa.DHA
MDA
DHAR
Pnet
APX
Gs
Proline
Asa
SOD
CAT
H2O2
Fv.Fm
tAsa
DHA
Cluster 1
Cluster 2
Cluster 3
PRO
Asa.DHA
-5
0
5
-1.0
-0.5
0.0
0.5
1.0
-6
-4
-2
0
2
4
